# Physical Distancing and Social Media Use in Emerging Adults and Adults During the COVID-19 Pandemic: Large-scale Cross-sectional and Longitudinal Survey Study

**DOI:** 10.2196/33713

**Published:** 2022-08-11

**Authors:** Thabo van Woudenberg, Moniek Buijzen, Roy Hendrikx, Julia van Weert, Bas van den Putte, Floor Kroese, Martine Bouman, Marijn de Bruin, Mattijs Lambooij

**Affiliations:** 1 Erasmus School of Social and Behavioural Sciences Erasmus University Rotterdam Rotterdam Netherlands; 2 Behavioural Science Institute Radboud University Nijmegen Netherlands; 3 Centre for Nutrition, Prevention and Health Services National Institute for Public Health and the Environment Bilthoven Netherlands; 4 Amsterdam School of Communication Research University of Amsterdam Amsterdam Netherlands; 5 Social, Health and Organisational Psychology University of Utrecht Utrecht Netherlands; 6 Corona Behavioural Unit National Institute for Public Health and the Environment Bilthoven Netherlands; 7 Erasmus School of History, Culture and Communication Erasmus University Rotterdam Rotterdam Netherlands; 8 Radboud Institute for Health Sciences Radboud University Medical Center Nijmegen Netherlands

**Keywords:** COVID-19, physical distancing, compliance, emerging adults, social media

## Abstract

**Background:**

Although emerging adults play a role in the spread of COVID-19, they are less likely to develop severe symptoms after infection. Emerging adults’ relatively high use of social media as a source of information raises concerns regarding COVID-19–related behavioral compliance (ie, physical distancing) in this age group.

**Objective:**

This study aimed to investigate physical distancing among emerging adults in comparison with adults and examine the role of using social media for COVID-19 news and information in this regard. In addition, this study explored the relationship between physical distancing and using different social media platforms and sources.

**Methods:**

The secondary data of a large-scale longitudinal national survey (N=123,848) between April and November 2020 were used. Participants indicated, ranging from 1 to 8 waves, how often they were successful in keeping a 1.5-m distance on a 7-point Likert scale. Participants aged between 18 and 24 years were considered emerging adults, and those aged >24 years were considered adults. In addition, a dummy variable was created to indicate per wave whether participants used social media for COVID-19 news and information. A subset of participants received follow-up questions to determine which platforms they used and what sources of news and information they had seen on social media. All preregistered hypotheses were tested with linear mixed-effects models and random intercept cross-lagged panel models.

**Results:**

Emerging adults reported fewer physical distancing behaviors than adults (β=−.08, t_86,213.83_=−26.79; *P*<.001). Moreover, emerging adults were more likely to use social media for COVID-19 news and information (b=2.48; odds ratio 11.93 [95% CI=9.72-14.65]; SE 0.11; Wald=23.66; *P*<.001), which mediated the association with physical distancing but only to a small extent (indirect effect: b=−0.03, 95% CI −0.04 to −0.02). Contrary to our hypothesis, the longitudinal random intercept cross-lagged panel model showed no evidence that physical distancing was not influenced by social media use in the previous wave. However, evidence indicated that social media use affects subsequent physical distancing behavior. Moreover, additional analyses showed that the use of most social media platforms (ie, YouTube, Facebook, and Instagram) and interpersonal communication were negatively associated with physical distancing, whereas other platforms (ie, LinkedIn and Twitter) and government messages had no or small positive associations with physical distancing.

**Conclusions:**

In conclusion, we should be vigilant with regard to the physical distancing of emerging adults, but the study results did not indicate concerns regarding the role of social media for COVID-19 news and information. However, as the use of some social media platforms and sources showed negative associations with physical distancing, future studies should more carefully examine these factors to better understand the associations between social media use for news and information and behavioral interventions in times of crisis.

## Introduction

### Background

In 2022, the COVID-19 pandemic is still ongoing in large parts of the world and, as of February, has been responsible for >381 million confirmed cases and >5.69 million deaths worldwide [1]. Given that most of the world’s population has not been vaccinated yet, alternative precautionary measures are still essential to contain the spread of the COVID-19 infection. Therefore, many countries have adopted behavioral interventions, of which physical distancing is one of the most widely adopted, persistent, pragmatic, and effective policies [2]. However, the effectiveness of such strategies depends heavily on the compliance of the population with desired behaviors [3]. It is therefore important to study and understand compliance with governmental behavioral interventions, such as physical distancing during the COVID-19 pandemic to design future interventions most effectively.

In times of crisis, such as these, people tend to rely heavily on media to understand the situation and make informed decisions about related behavioral guidelines [4,5]. According to cultivation theory [6], content across the entire media landscape breeds a widespread meaning among the audience. The theory proposes that the more media-provided information the people consume, the greater the likelihood that their perceptions of reality align with that depicted in the media landscape. This cultivation process is driven by both mainstreaming and resonance, that is, different opinions and world viewpoints will move to align their opinions with the mediated content, and simultaneously, the mediated content becomes more relatable and relevant to media consumers. This means that people’s perceptions and intentions will ultimately become similar to what is portrayed in the media landscape [6]. Therefore, the more the media emphasizes on the severity of the situation and the importance of physical distancing, the more likely it is that people will change their behavior.

Moreover, social cognitive theory [7] explains how a single media message can affect the behavior of people. This theory explains that people create cognitive schemas based on first-person experiences and observational learning. A large part of observational learning occurs through media exposure [8], meaning that people see and learn from others about COVID-19 and the counteractive measures via media exposure and adjust their perceptions and behavior accordingly [9]. This means that people learn how to behave from others portrayed in the media during the COVID-19 pandemic.

However, the nature and content of media messages in social media and mass media have different effects on people’s perceptions and behavioral intentions during a crisis [10]. Although cultivation processes occur in both traditional media and social media [11], the nature and content of the message differ between the 2 forms of media. The immediacy of social media and the direct access to an unprecedented amount of content allows for less controlled and more fragmented view of the crisis [12-14]. Therefore, the process of mainstreaming and resonance is less likely to occur, and the importance of physical distancing will be less cultivated among social media users for COVID-19 news and information.

In addition, social media depicts more ambivalent messages about COVID-19 than traditional mass media does, contains more rumors or questionable information [12,15], is more subjective to algorithms that mediate and facilitate content promotion [16], and is more likely to only reach and circulate in subgroups of users in so-called echo chambers [17]. In general, people who use social media to inform themselves about COVID-19 will observe a broader range of ideas and behaviors on the web than those who only use traditional media. Therefore, it is less clear what normative behavior is during a crisis, and people are less likely to change their behaviors to comply with governmental behavioral interventions. This difference in behavioral change between social media users and nonusers has been observed in previous crises. For example, research on news consumption after the Great East Japan Earthquake in 2011 has demonstrated that mass media has a positive effect on people’s perceptions of a crisis and the subsequent increased behavior change (ie, boosting civic communications, taking altruistic actions, and preparing for future crises). Social media showed only limited or no change in perceptions and behavioral intentions [10]. For the current crisis, this would mean that people who use social media to inform themselves about the crisis are less likely to change their behavior and, therefore, less likely to be physically distant from others.

This difference in news and information consumption and associated compliance with behavioral regulations is problematic when particular subgroups of the population rely more heavily on social media for news and information on COVID-19. In particular, young people differ substantially in news consumption compared with older generations. They are more attracted to social media as a source of news and information [18-20]; therefore, it seems likely that younger people also consume relatively more COVID-19 news and information via social media than adults do [21-23]. As a result, the subgroup of young people, on average, would be less likely than adults to change their behavior and comply with behavioral regulations. Aside from a lower health risk when exposed to the coronavirus [24-26] and a stronger need to socialize with others [27] (A Orben, unpublished data, August 2020), this difference in the consumption of COVID-19 news and information might be important in understanding compliance of young people with behavioral regulations. That is, using social media for COVID-19 news and information might explain why young people are less often maintaining a physical distance from others.

A review of studies on protective behaviors during several pandemics before the COVID-19 crisis showed that older people have a higher chance of adopting relevant protective behaviors [28]. Contemporary research on COVID-19 corroborated this finding and showed that younger people engage less often in protective behaviors, such as physical distancing, than do older people. For example, a cross-sectional survey in the United States showed that adherence to distancing behaviors of young people aged between 18 and 24 years was considerably less than that of adults [29]. Similarly, other studies showed a linear increase in age with a range of protective behaviors, including physical distancing [30,31].

In this study, we are particularly interested in young people aged 18 years to their late 20s, termed *emerging adults* [32]. As these emerging adults grow as autonomous adults, they become more independent media consumers and less influenced by their parents. This is in contrast to the vast majority of children and adolescents who live with their parents and the associated influence of living with their parents on their media use [33,34]. A better understanding of the role that social media plays in compliance with behavioral interventions in emerging adults is valuable knowledge for governments, as this will help them better communicate behavioral regulations to all its citizens, boost the effectiveness of comparable behavioral interventions, and ultimately save lives.

### This Study

This study investigated the differences between emerging adults and adults in terms of their physical distancing behavior while considering the role of using social media for COVID-19 news and information. On the basis of the theoretical framework and related empirical findings, we preregistered the following hypotheses: physical distancing is lower in emerging adults than in adults (H1), and the effect of age on physical distancing is mediated by the use of social media for COVID-19 news and information (H2). More specifically, we anticipated that age would negatively predict using social media for COVID-19 news and information (H2a) and using social media for COVID-19 news and information would negatively predicts physical distancing (H2b). This study further investigated the directionality of the association between physical distancing and social media use in a longitudinal sample.

In addition, to gain more insight into specific social media use, we performed exploratory research on a subsample of participants who were presented with an additional module of the questionnaire. These questions examined the use of different social media platforms and sources of messages consumed on social media. Specifically, these nonpreregistered analyses examined the association of physical distancing with (1) the most often used social media platforms (ie, Facebook, Twitter, Instagram, YouTube, and LinkedIn) and (2) the sources presented on the platform (ie, government, national news, regional news, personal communication, or another source).

## Methods

### Ethics Approval

We used secondary data from a large-scale national longitudinal study conducted by the Dutch National Institute for Public Health and the Environment. Participants provided informed consent before the start of the first survey. The data that we received did not contain any identifiable information. Therefore, this study did not require to be reviewed by an institutional review board. The study design, hypotheses, measured variables, and plan of analysis were preregistered before gaining access to the data and can be found on the Open Science Framework page of this study [35].

### Participants and Procedure

Participants were recruited via 25 municipal health offices (Gemeenschappelijke Gezondheidsdienst) to participate in the national survey (N=124,580). Participants were asked to fill out 1 or more questionnaires during 8 waves of data collection between April and November 2020. During this period, COVID-19 was highly prevalent in the Netherlands, ranging from 0.47 to 57.87 daily new cases per 100,000 inhabitants, and various preventive measures were in effect. Some of the measures that were continuously communicated were to keep a physical distance from others (1.5 m), to not shake hands and to wash hands often, to sneeze and cough in the armpit, and to work from home as much as possible. Initially, participants received a questionnaire every 3 weeks, and after the fifth wave, the interval was increased to 6 weeks (Table 1).

For each wave, the survey was divided into 3 subcomponents, and each participant received 1 of these 3 subcomponents per wave. As a result, a subset of participants received questions relevant to this study. Also, the preregistered exclusion criteria were used to exclude participants aged <18 years and participants for which the control variables were missing. This resulted in an analytical sample of 123,848 adults aged >17 years (34.11% men) who participated in 1 wave (n=47,708, 38.5%) or multiple waves (n=76,140, 61.5%). The participants in the longitudinal sample participated in 2 and 8 waves (mean 5.36, SD 2.14). As this study used existing data, no a priori sample size calculation was performed. Given the sample size, we did not anticipate problems with the statistical power. For each analysis, the number of included participants and observations is reported.

**Table 1:** Number of participants and dates of measurements per wave.

Wave	Number of participants	Between dates
1	65,572	April 17, 2020, to April 24, 2020
2	52,847	May 7, 2020, to May 12, 2020
3	63,773	May 27, 2020, to June 1, 2020
4	50,200	June 17, 2020, to June 21, 2020
5	50,366	July 8, 2020, to July 12, 2020
6	61,361	August 19, 2020, to August 23, 2020
7	47,670	September 30, 2020, to October 4, 2020
8	63,989	November 11, 2020, to November 15, 2020

### Measures

#### Physical Distancing

For each wave, participants first answered the question “In the past 7 days, how often were you with a group of four or more people with whom you do not live in 1 house? For example, at work, at the park, on the street with neighbors, or at a birthday” on a scale ranging from *never* (1) to *more than 20 times* (7). Participants who were, at least one time, with a group of 4 or more people in the last week were asked the follow-up question “In the past 7 days, how often were you successful in always keeping a physical distance of 1.5 meters from these people” and asked to respond on a Likert scale ranging from *never* (1) to *always* (7). The score on this scale for each wave was used as a measure of physical distancing. The higher the score on the variable, the more successful the participant was in maintaining physical distance in the past week (mean*_grand_* 4.34, SD*_grand_* 1.58).

#### Age

The participants indicated the category according to their age group (Table 2). As only 0.54% (669/123,848) of the participants were in the eighth category (≥85 years), categories 7 and 8 were merged. Dummy coding was used to create a contrast between the emerging adults (n=6648) and older age categories (n=117,200). To further investigate differences between the age categories, reversed Helmert contrast coding was used to contrast the age category with all higher age categories combined, starting with the emerging adult category.

**Table 2:** Age in categories (N=123,848).

Answer	Age (years)	Label	Participants, n (%)
3	18-24	Emerging adults	6648 (5.37)
4	25-39	Early career	31,724 (25.62)
5	40-54	Midcareer	34,692 (28.01)
6	55-69	Late career	33,476 (27.03)
7-8	≥70	Retired	17,308 (13.98)

#### Social Media Use

Each wave, a subset of participants answered the question “In the past 7 days, which of these sources did you use to get information and news about the coronavirus?” Participants could respond by selecting 1 or more media sources from the given list. One such source was *social media*. A dummy variable *Social Media* was created to compare whether participants used social media (0.5, n_observations_=33,941) or did not (−0.5, n_observations_=81,008) for COVID-19 news and information per wave.

#### Social Media Platforms and Sources

In waves 2 and 4, a subset of the participants (n=18,047) received the module with more extensive questions regarding social media use. In these questions, participants indicated how many days of the past week they had used the following platforms for COVID-19 news and information: Facebook, Twitter, Instagram, YouTube, and LinkedIn. For each indicated social platform, participants were also asked to select 1 or multiple sources presented on the platform: government, national news, regional news, personal communication, or other sources.

#### Control Variables

To control for potential differences in physical distancing, all analyses were controlled for participant sex. In addition, the wave was added as a covariate to control for potential changes in behavior and context over time. As not all participants filled out the questions during the same wave, it is important to control this temporal context. During the measurements, the number of infected people was initially high and decreased during the summer but increased again after the fifth wave. In addition, the regulations changed regularly, and the overall sentiment might have changed as well. A linear wave variable would not reflect this trend; therefore, we have tested several other shapes that would fit the observed data [36]. The wave-transformed variable with the best fit to the observed data was selected. Specifically, the wave variable was centered on wave 5, and the absolute values were used to create a v-shape. The standardized effect of the transformed variable was higher and explained more variance (*R*^2^_marginal_=0.031, β=0.18) than the linear wave variable (*R*^2^*_marginal_*=0.0002, β=−0.05).

### Strategy of Analysis

We preregistered the intention to use Bayesian statistics to test the hypothesis. However, all analyses had to be performed on a secured remote desktop, and the possibilities of running extensive computations on this large data set were limited. Therefore, multivariate mixed effects models were run by using the *lme4* package [37] in R (R Foundation for Statistical Computing) [38]. SE, CIs, and *P* values were computed using the Satterthwaite approximation [39], and CIs not including 0 or *P*<.05 were considered statistically significant. Effect sizes were used to determine the direction and relative strength of the parameter, and parameter importance was determined based on the improved model fit.

In the mixed effects models, sex and wave were added as covariates, and random intercepts were included per participant. According to this hypothesis, the predictor was substituted for the variable of interest. To test the mediation for H2, a multilevel mediation model from the *mlma* package was used [40]. In addition, to determine the cross-lagged effects between physical distancing and using social media as a source, Random intercept cross-lagged panel models [41] were used to distinguish between-person (stable time-invariant traits) and within-person (in-person changes over time) associations. The cross-lagged paths were used to assess the directionality between using social media and physical distancing between current and subsequent waves while controlling for stability traits between waves and covariance within waves. All correlations at each wave, stability, and cross-lagged paths were restricted to be the same, resulting in 1 parameter estimate per path type.

In contrast to the preregistration, the weather conditions were not included as covariates because the exact dates of filling out the questionnaires were not included in the data set. We have tried to include the average weather conditions per wave, but this variable had too much collinearity with the wave variable, making the models unidentifiable. Moreover, the hypotheses on well-being are not reported in this paper because of an overlap with another group of researchers working with the same data set. The planned analyses are still part of the supportive materials for the Open Science Framework. Finally, 6 waves of data were available at the time of registration. Subsequently, 2 additional waves of data were gathered, which were added to the data set.

On top of the preregistered analyses, 2 exploratory analyses were performed on the subsample of the participants that received the module on the use of specific social media platforms (ie, Facebook, Twitter, Instagram, YouTube, and LinkedIn) and different sources that appear on these platforms (ie, governmental, national news, regional paper, personal post, or other sources). The 2 mixed effects models were specified similarly to the model to test the first hypothesis. In the first model, the number of days per week that participants used social media platforms for COVID-19 news and information were entered as predictors, and the age variable was treated as a covariate. In the second exploratory model, social media variables were again excluded, and dummy variables per source were used to determine whether participants were exposed to a specific source on social media.

## Results

### Physical Distancing

The linear mixed-effects model that was used to test the first hypothesis consisted of a random structure in the form of random intercepts per participant and a fixed structure explaining 4% of the variance in physical distancing (marginal *R*^2^). Both fixed and random effects explained 50% of the variance (conditional *R*^2^). The intraclass correlation coefficient of the random effect participant was 0.48, indicating that approximately half of the variance was explained by other observations on the outcome variable within the same participant.

**Figure 1:**
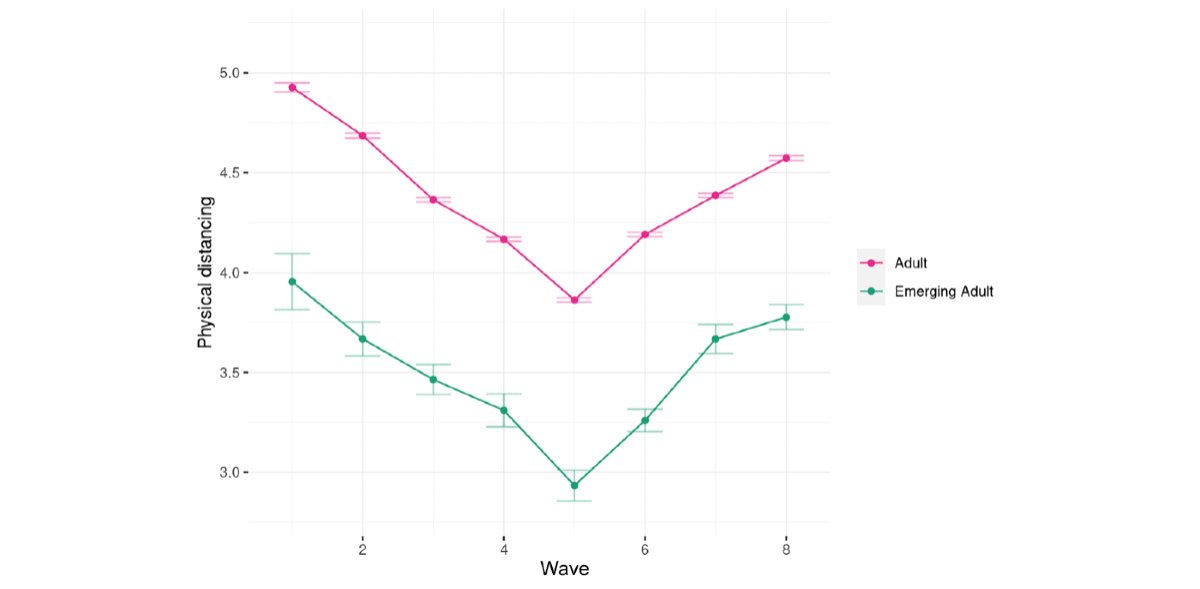
Physical distancing in emerging adults and adults over the eight waves.

The planned contrast indicated that emerging adults (mean_marginal_ 3.48, SE_marginal_ 0.03) maintained physical distance from others less often than the older participants (mean_marginal_ 4.37, SE_marginal_ 0.01; Figure 1).

The standardized effect size suggested that the effect of age was less important than that of the covariate wave (Table 3). However, the model fit and explained variance of the model (Akaike information criterion [AIC]=693,681 and Bayesian information criterion [BIC]=693,742) were better than the model fit without the emerging adult variable (*R*^2^_marginal_=0.03, AIC=694.394 and BIC=694.444; *χ^2^*_1_=714.9; *P*<.001). This indicated that although the effect of age could be considered small, the variable still contributed to explaining physical distancing behavior.

**Figure 2:**
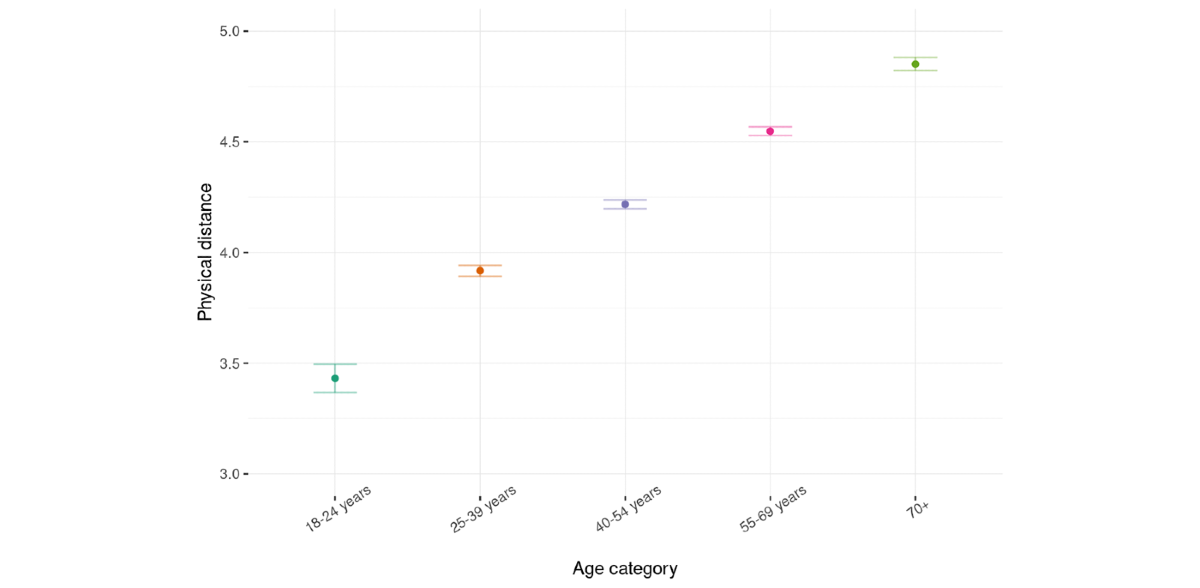
Physical distancing per age category.

In a nonpreregistered additional analysis, we further investigated the differences in physical distancing between age categories. Therefore, the dichotomous *emerging adult* variable was substituted for multiple contrasts of the categorical age variables, as measured in the project. This variable increased the marginal *R*^2^ of the model to 6% and indicated that with an increase in the age category, people practiced physical distancing more often (Figure 2). Together, these analyses provide support for hypothesis 1 that physical distancing is lower in emerging adults than in adults.

**Table 3:** Multivariate linear mixed-effects model predicting physical distancing behavior (n=70,629; number of observations=185,208; participant intraclass correlation coefficient=0.48; marginal R^2^=0.04; conditional R^2^=0.50).

Variable	B (SE)	95% CI	β	*t* test (*df*)	*P* value
Intercept	3.44 (0.02)	(3.41 to 3.48)	.00	194.46 (98,929.46)	<.001
Emerging adult	−0.89 (0.03)	(−0.96 to −0.82)	−.08	−26.79 (86,213.83)	<.001
Sex	0.12 (0.01)	(0.10 to 0.14)	.03	10.04 (65,587.82)	<.001
Wave	0.29 (0.00)	(0.29 to 0.30)	.18	96.81 (148,077.18)	<.001

### Using Social Media for COVID-19 News and Information

The second hypothesis investigated the role of using social media for COVID-19 news and information in physical distancing. The related social media use question was asked in waves 3, 5, and 8 in a subsample of participants (n=17,714, n_observations_=38,423). A social media use dummy was added to the model used in H1 (R^2^_marginal_=0.03, R^2^_conditional_=0.48, intraclass correlation coefficient_participant_=0.48). A significant social media use parameter indicated that those who used social media (mean_marginal_ 3.96, SE_marginal_ 0.06) showed slightly less physical distancing behavior than those who did not use social media for COVID-19 news and information (mean_marginal_ 4.03, SE_marginal_ 0.06). In the same model, a significant emerging adult parameter indicated that after controlling for social media use, emerging adults kept physical distance less often than did adults (Table 4). Again, the standardized effect sizes for both social media use and emerging adults were small. An improved model fit indicated that both the social media use variable (full model: AIC=108,886, BIC=108,944; model without social media use predictor: AIC=108,900, BIC=108,950; *χ^2^*_1_=16.3; *P*<.001) and emerging adult variables (model without social emerging adult predictor: AIC=108,942, BIC=108,991; *χ^2^*_1_=57.7; *P*<.001) contributed to explaining physical distancing behavior.

Next, a mixed effects logistic regression model was used to test whether emerging adults were more likely to use social media for news and information on COVID-19. The model (*R*^2^_marginal_=0.05, *R*^2^_conditional_=0.76) showed a significant emerging adult parameter (B=2.48; SE 0.10; Wald=23.66; *P*≤.001). Emerging adolescents were 11.93 (95% CI 9.72-14.65) times more likely to use social media for COVID-19 news and information than adults did. This means that there is a stronger preference to use social media for COVID-19 news and information among emerging adults than among adults.

Finally, a mixed effects mediation model was used to dissociate the direct association between the emerging adult variable and physical distancing from the indirect association mediated by social media for COVID-19 news and information. The model showed that the total effect (β=−.91; 95% CI 1.06 to −0.77; *P*<.001), direct effect (β=−.88; 95% CI 1.04 to −0.74; *P*<.001), and indirect effect (β=−.03; 95% CI 0.04 to −0.02; *P*<.001) were all significant. However, the indirect effect was substantially smaller than the direct effect, and we concluded that there is a partial, but limited, mediating path of using social media for COVID-19 news and information. Therefore, using social media for COVID-19 news and information can only marginally explain why physical distancing is lower among emerging adults than among adults.

**Table 4:** Multivariate linear mixed-effects model predicting physical distancing behavior (n=17,714; number of observations=38,423; intraclass correlation coefficient of participants=0.47; marginal R^2^=0.03; conditional R^2^=0.48).

Variable	B (SE)	95% CI	β	*t* test (*df*)	*P* value
Intercept	3.56 (0.06)	(3.45 to 3.68)	.00	60.24 (13,774.81)	<.001
Social media use	−0.10 (0.02)	(−0.15 to −0.05)	−.02	−4.03 (28,459.80)	<.001
Emerging adult	−0.87 (0.11)	(−1.09 to −0.65)	−.06	−7.60 (12,566.76)	<.001
Sex	0.11 (0.03)	(0.06 to 0.17)	.03	3.94 (11,431.76)	<.001
Wave	0.27 (0.01)	(0.26 to 0.29)	.16	34.67 (21,764.25)	<.001

### Determining Directionality

An additional analysis investigated the directionality of the effect of using social media on physical distancing and vice versa. This analysis used a subset of the participants (n=7325) in the last 4 waves because then the social media question was presented in 4 subsequent waves to the same participants. In addition, the sex and age groups of the participants were added as covariates in the model. The restricted random intercept cross-lagged panel model (*χ^2^*_35_=296.9; *P*≤.001; comparative fit index=0.987, Tucker–Lewis index=0.984, root mean square error of approximation=0.032, and standardized root mean square residual=0.025) showed a negative relationship between social media use and physical distancing (Table 5). This means that there was a small negative between-person association between physical distancing and using social media for COVID-19–related news and information. In addition, both stability paths were significant, indicating that the values of both variables were predicted by the value of the previous wave. Most interestingly, a small negative cross-lagged effect of physical distancing on social media use, but no effect of social media on physical distancing, was observed. This indicates that using social media for COVID-19 news and information did not affect physical distancing in the subsequent wave. By contrast, those who maintained physical distance less often were more likely to use social media as a source in the subsequent wave. However, the standardized effect size was very small.

**Table 5:** Random intercept cross-lagged panel model of physical distancing and social media (n=7324).

Variable	B (SE)	95% CI	β	*z* score	*P* value
W5 correlation	−0.01 (0.01)	(−0.02 to 0.00)	−.03	−2.21	.03
Distance → social media	0.00 (0.00)	(−0.01 to 0.00)	−.02	−2.14	.03
Social media → distance	−0.06 (0.04)	(−0.14 to 0.02)	−.02	−1.40	.16
Distance → distance	0.12 (0.01)	(0.10 to 0.14)	.12	10.94	<.001
Social media → social media	0.11 (0.01)	(0.09 to 0.14)	.11	9.9	<.001
Correlated change W6-8	0.00 (0.00)	(−0.01 to 0.01)	.00	0.33	.74
Between-person correlation	−0.01 (0.01)	(−0.03 to −0.00)	−.04	−2.54	.01

### Differences in Social Media Platforms and Sources of Information on Social Media

In the last 2 analyses, we further explored the differences between several social media platforms and the sources that appear on these platforms for COVID-19 news and information. The explorations were performed in a subsample of participants who received the extensive social media module in waves 2 and 4 (n=9992 and n_observations_=12,456). Facebook was the most frequently used platform (5274/12,456, 42.34%), whereas all other platforms were used between 15.7% (1995/12,456) and 11.03% (1374/12,456) of the time. When a social media platform was used, Facebook (n=5274; mean 4.91, SD 2.36), Instagram (n=1881; mean 4.49, SD 2.44), and Twitter (n=1786; mean 4.68, SD 2.38) were used for more than half of the days per week for COVID-19 news and information. LinkedIn (n=1955; mean 3.16, SD 2.12) and YouTube (n=1374; mean 2.81, SD 2.07) were used for fewer days per week for COVID-19 news and information.

The first linear mixed-effects model (*R*^2^_marginal_=0.07, *R*^2^_conditional_=0.89) investigated the association between physical distancing and the number of days per week during which different social media platforms were used for COVID-19 news and information (Table 6).

**Figure 3:**
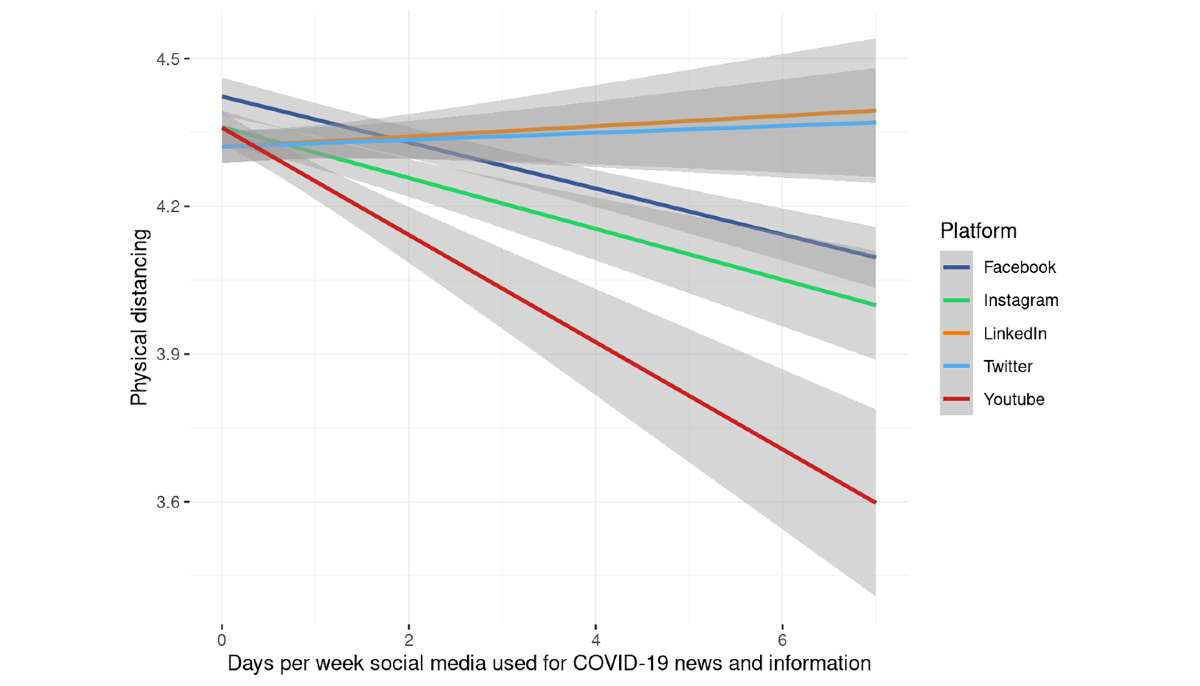
Associations between the number of days spent using different social media platforms and physical distancing.

The results of the model showed that some platforms had no association or a slightly positive association with physical distancing (ie, Twitter and LinkedIn), whereas others had a negative association (ie, Facebook, Instagram, and YouTube; Figure 3).

Potentially, differences in associations emerged because various information sources were portrayed on different platforms. Therefore, we further investigated the sources of COVID-19 news and information on the social media platforms used by participants. Governmental (6511/12,456, 52.3%), national news (6429/12,456, 51.6%), and personal communication (7237/12,456, 58.1%) were the most common sources on social media platforms. Regional news (3036/12,456, 24.4%) and other sources (1454/12,456, 11.5%) were used less frequently for COVID-19 news and information.

In the second linear mixed-effects model, the social media platform variables were substituted for a dummy variable per source, contrasting seeing a source (0.5) on social media versus not seeing a source on social media (−0.5). The model (*R*^2^_marginal_=0.06, *R*^2^_conditional_=0.53) showed that being exposed to governmental sources had a distinctly small positive association, compared with the other sources that had no or a small negative association with physical distancing (Table 7). Together, these 2 exploratory analyses suggest that associations between physical distancing and using social media for COVID-19 news and information are less straightforward. Depending on the social media platform that people used and the sources they were exposed to on social media, the associations varied in effect size and direction.

**Table 6:** Multivariate linear mixed-effects model predicting physical distancing behavior (n=9992; number of observations=12,456; intraclass correlation coefficient of participants=0.48; marginal R^2^=0.04; conditional R^2^=0.50).

Variable	B (SE)	95% CI	β	*t* test (*df*)	*P* value
Intercept	4.01 (0.07)	(3.88 to 4.15)	.00	57.72 (9734.55)	<.001
Facebook	−0.04 (0.01)	(−0.05 to −0.03)	−.06	−6.06 (11,930.58)	<.001
Twitter	0.02 (0.01)	(0.00 to 0.04)	.02	2.17 (11,380.53)	.03
Instagram	−0.02 (0.01)	(−0.04 to 0.00)	−.02	−2.38 (12,429.39)	.02
YouTube	−0.09 (0.01)	(−0.12 to −0.07)	−.06	−6.36 (12,443.79)	<.001
LinkedIn	0.04 (0.01)	(0.01 to 0.06)	.03	2.94 (12,262.32)	.003
Sex (male)	0.12 (0.04)	(0.05 to 0.19)	.03	3.43 (9871.87)	<.001
Wave	−0.57 (0.03)	(−0.62 to −0.52)	−0.16	−20.88 (6389.34)	<.001
Emerging adult	−1.00 (0.14)	(−1.27 to −0.73)	−0.07	−7.36 (9631.78)	<.001

**Table 7:** Multivariate linear mixed-effects model of social media sources predicting physical distancing behavior (n=5986; number of observations=7221; intraclass correlation coefficient of participants=0.48; marginal R^2^=0.04; conditional R^2^=0.53).

Variable	B (SE)	95% CI	β	*t* test (*df*)	*P* value
Intercept	3.85 (0.08)	(3.68 to 4.01)	.00	46.39 (5994.70)	<.001
Government	0.10 (0.03)	(0.05 to 0.16)	.05	3.68 (7178.46)	<.001
National news	−0.05 (0.03)	(−0.11 to 0.01)	−.02	−1.73 (7106.50)	.08
Regional news	−0.04 (0.03)	(−0.11 to 0.03)	−.01	−1.14 (7077.41)	.25
Personal communication	−0.08 (0.02)	(−0.13 to −0.04)	−.05	−3.86 (7162.97)	<.001
Other	−0.10 (0.04)	(−0.18 to −0.03)	−.03	−2.74 (7085.96)	.006
Sex (male)	0.05 (0.05)	(−0.05 to 0.14)	.01	0.96 (5862.23)	.34
Wave	−0.59 (0.04)	(−0.66 to −0.52)	−.16	−16.35 (3694.48)	<.001
Emerging adult	−1.05 (0.15)	(−1.35 to −0.75)	−.09	−6.93 (5691.54)	<.001

## Discussion

### Principal Findings

This study investigated differences between emerging adults and adults in terms of physical distancing. Moreover, the role of using social media for COVID-19 news and information was investigated by examining its mediating role and to what extent different social media platforms and sources relate to physical distancing. These questions were addressed in a longitudinal panel study of a large sample of Dutch adults conducted between April and November 2020. On the basis of our findings, 3 main conclusions can be drawn.

First, our findings demonstrate that the physical distancing behavior is lower in the group of emerging adults than in the group of adults. Moreover, we believe that physical distancing increases with age as the exploration with nondichotomous age categories implies. This finding was in line with previous studies that reported that emerging adults or younger participants, on average, maintain physical distance less often than adults [29,31]. One potential explanation lies in psychosocial models such as the health belief model [42] and protection motivation theory [43]. Given the lower personal health risks for younger people, the perceived vulnerability, severity, and perceived benefits of physical distancing might be lower, whereas the cost and barriers to compliance would be higher, giving up more of social daily life [27,31].

The second conclusion is that using social media for news and information on COVID-19 is negatively related to physical distancing behavior, irrespective of age. Moreover, the emerging adults in our study were more likely to use social media for COVID-19 news and information, and social media played a small role in physical distancing behaviors. However, because the indirect relationship was trivially small compared with the direct relationship, we consider social media use only as a very limited, not meaningful, explanation of emerging adults’ lower physical distancing behavior.

Moreover, the longitudinal panel model showed no support for the direction of social media use, leading to lower physical distancing in the subsequent wave. Rather, the analysis showed a significant, albeit small, cross-lagged path between physical distancing and future social media use. A potential explanation is selective attention to COVID-19 news and information that affirms their current beliefs about COVID-19 and avoids media content that is dissonant with their behavior [44,45], similar to political news seeking [46]. This would mean that those who disagree with the prevailing measures turn away from other types of sources such as television, governmental websites, and newspapers when they seek information and news about the coronavirus, as the portrayed images are not in line with their beliefs. Therefore, using social media for COVID-19 news and information can be seen as a sign of noncompliance and not as a source of noncompliance to the prevailing measures. However, the observed effect in this study was so small that at this stage, we are not in a position to draw firm conclusions, and further investigation is warranted.

Finally, the explorations in this study suggest that social media use is not always bad for physical distancing behaviors, with some platforms showing small positive relations with physical distancing. Moreover, the types of sources portrayed in these social media messages seem to relate to physical distancing. We can draw tentative conclusions that users looking for COVID-19 news and information on LinkedIn and Twitter were more likely to adhere to physical distancing measures, albeit with relatively weak associations. Similarly, the use of social media posts from governmental sources was related to greater physical distancing, whereas web-based personal communication seemed to be related to less physical distancing. Overall, it should be noted that the strength of the observed associations between social media use and physical distancing was relatively low or even nonsignificant, such as for national and regional news sources on social media.

### Strengths and Limitations

In the literature on COVID-19, an impressive number of studies have investigated the impact of the virus and its corresponding regulations. Some studies have focused on student samples but overlooked going beyond young people who attend postsecondary education or comparing this age group to adults. In this study, we had the opportunity to fill this gap by using a very large sample of emerging adults and adults. In addition, a substantive subset was part of a longitudinal sample, enabling us to investigate the relationships over time and sensitize the directionality of relationships. By using both multivariate mixed effects models and random intercept cross-lagged panel models, we were able to control for the clustering of data with each participant that responded multiple times and investigated the directionally of the studies association. Furthermore, open-science practices were used, in which the hypotheses and analyses were preregistered before the analyses were performed, and the scripts used are publicly available.

This research also has some limitations that must be considered when interpreting the findings, which can be addressed in future research. For example, a crude measurement of participants’ age was used. As secondary data were used, this study had no control over the questions being asked or the data being stored. The survey was carried out with much attention paid to the privacy of the participants. To reduce the traceability of the participants, the age variable was measured in categories. We encourage these anonymization efforts, but they might have made the estimated parameters less precise, and another level of detail could have been achieved by having the exact age of the participants. Future large-scale projects could address this issue by creating synthetic data sets before analyzing the data to retain the privacy of the participants [47].

In addition, physical distancing was measured through retrospective self-report. Participants indicated in each wave how often they had maintained physical distance from others in the preceding 7 days. Considering all potential biases (eg, recall bias, primacy and recency bias, and social desirability), it is conceivable that the reported behavior deviates from the objective physical distancing behavior. However, we do not believe that the effects of potential biases may be different for different age groups. Related to this is the measurement of sources used for information and news on COVID-19. Participants responded by selecting several types of media from the provided list. The actual amount and specific content seen on social media or other sources could not be derived in this study. One way of obtaining more detailed information in large-scale studies would be to ask participants to donate logging data of the used social networking sites (eg, cookies or browser history) or ask participants to install a mobile sensing app to collect media use and physical distancing behaviors [48,49].

Finally, the size of the sample also warrants some caution in the context of null hypothesis significance testing because even tiny effects can reach the preregistered critical value of *P*<.05. As a result, the question arises of whether the significant effect is big enough to be concerned about. In our analyses, we used standardized effect sizes representing a 1 SD increase on the Likert scale measuring physical distancing. However, as the answers on the Likert scale do not form an absolute continuous scale, a quantifiable interpretation of the size of the significant effects is not straightforward. We have tried to indicate whether we deem the effect meaningful by examining an increased model fit of a particular variable. However, at the same time, the large sample size eliminates the argument of insufficient power to detect an effect and make a type 2 error. This gives us more confidence in deciding that when an effect is not statistically significant, it is highly likely to be absent. However, other arguments regarding why the hypotheses can be falsely rejected remain applicable to this study.

### Conclusions

Our study indicates a substantive gap between emerging adults and adults in physical distance behavior during the COVID-19 pandemic and yet yields a nuanced view on emerging adulthood and the role of social media. Given the overall increase with age, we cannot make firm conclusions that the group of emerging adults should be seen as a particularly problematic group in itself but rather that the older people become, the more often they comply with physical distancing measures. Moreover, although using social media for COVID-19 news and information is negatively related to physical distancing behavior, it does not seem to be an important factor in explaining why emerging adults comply less with the behavioral measures, nor does it lead to changes in physical distancing behavior over time. Finally, there are differences between the various social media platforms and sources, with some platforms and sources showing negative associations and other platforms showing positive to no associations with physical distancing. However, we should be cautious in assuming that these social media affect behaviors because they may very well be indicators of selective exposure to social media that match one’s physical distancing behaviors.

## Abbreviations

AIC: Akaike information criterion
BIC: Bayesian information criterion
